# Effects of 1,25-Dihydroxyvitamin D3 on the Inflammatory Responses of Stromal Vascular Cells and Adipocytes from Lean and Obese Mice

**DOI:** 10.3390/nu12020364

**Published:** 2020-01-30

**Authors:** Chan Yoon Park, Tae Yeon Kim, Ji Su Yoo, Yeonkyung Seo, Munkyong Pae, Sung Nim Han

**Affiliations:** 1 Department of Food and Nutrition, College of Human Ecology, Seoul National University, Seoul 08826, Korea; violin925@snu.ac.kr (C.Y.P.); ppeace114@snu.ac.kr (T.Y.K.); dbwltn2349@snu.ac.kr (J.S.Y.); yeon1011@snu.ac.kr (Y.S.); 2 Department of Food and Nutrition, College of Human Ecology, Chungbuk National University, Cheongju 28644, Korea; mpae@chungbuk.ac.kr; 3 Research Institute of Human Ecology, Seoul National University, Seoul 08826, Korea

**Keywords:** obesity, 1,25-dihydroxyvitamin D, stromal vascular cell, adipose tissue inflammation, vitamin D supplementation, macrophage infiltration

## Abstract

Vitamin D status has been implicated in obesity and adipose tissue inflammation. In the present study, we explored the effects of dietary vitamin D supplementation on adipose tissue inflammation and immune cell population, and the effects of in vitro 1,25-dihydroxyvitamin D3 (1,25-(OH)2D3) treatment on pro-inflammatory cytokine production by stromal vascular cells (SVCs) and adipocytes in lean and high-fat diet-induced obese mice. The results show that epididymal fat *Mcp-1* and *Rantes* mRNA levels, which were higher in obese mice compared with lean mice, were significantly down-regulated by vitamin D supplementation. While obese mice had higher numbers of macrophages and natural killer (NK) cells within adipose tissue, these remained unaltered by vitamin D supplementation. In accordance with these in vivo findings, the in vitro 1,25(OH)2D3 treatment decreased IL-6, MCP-1, and IL-1β production by SVCs from obese mice, but not by adipocytes. In addition, 1,25(OH)2D3 treatment significantly decreased *Tlr2* expression and increased mRNA levels of *Iκba* and *Dusp1* in SVCs. These findings suggest that vitamin D supplementation attenuates inflammatory response in adipose tissue, especially in SVCs, possibly through inhibiting NF-κB and MAPK signaling pathways in SVCs but not by the inhibition of macrophage infiltration.

## 1. Introduction

The prevalence of obesity and related disorders are increasing worldwide. Adipose tissue expansion or fat accumulation in the liver, observed with obesity, activates the pro-inflammatory signaling pathway, which mediates pathogenesis of metabolic disorders, such as type 2 diabetes and nonalcoholic fatty liver disease (NAFLD) [1,2,3,4]. Hypovitaminosis D is often observed in obese humans [5]. New evidence suggests that low serum vitamin D may cause and exacerbate insulin resistance and NAFLD [6,7,8]. Several in vitro studies have shown that 1,25-dihydroxyvitamin D3 (1,25(OH)2D3) treatment could alleviate inflammatory responses in mouse or human adipocytes [9,10].

Adipose tissue, composed of adipocytes and non-adipocytes known as stromal vascular cells (SVCs), functions not only as an energy reservoir but also as a mediator for immune and inflammatory responses by releasing more than 260 proteins [11,12]. Obesity leads to hypertrophic adipose expansion, which triggers the recruitment of inflammatory immune cells and the secretion of pro-inflammatory cytokines. The number of macrophages in adipose tissue increases substantially during the course of obesity, composing half of the total adipose immune cells [2]. The production of inflammatory mediators, including monocyte chemoattractant protein-1 (MCP-1), interleukin (IL)-6, IL-1β, and tumor necrosis factor (TNF)α, increases with obesity since macrophages contribute to the production of them [13,14]. Besides macrophages, other immune cells also contribute to low-grade inflammation in an obese state. It has been reported that CD8+ T cells could contribute to macrophage infiltration [14,15] and the population of CD4+ T cells, both Th1 and Th17 cells, could be increased in adipose tissues from obese mice or obese humans [16,17,18]. The inflammatory response in adipose tissue is also triggered by pattern recognition receptors (PRRs), such as toll-like receptors (TLRs) and nucleotide-binding oligomerization domain proteins (NODs) [19]. TLR2 and TLR4 are activated not only by a pathogen-associated molecular pattern (PAMP) but also by fatty acids released from lipolysis in adipocytes [14,20]. Therefore, obesity could increase TLR2 or TLR4 expression in adipose tissue and activate the nuclear factor-κB (NF-κB) and mitogen activated protein kinases (MAPKs) signaling pathways, leading to chronic inflammation [21,22,23,24].

The identification of the vitamin D receptor (VDR) in adipocytes and hematopoietic cells suggests that 1,25(OH)2D can involve in diverse localized effects including lipogenesis, innate and adaptive immune responses, or inflammation within adipose tissue [25]. Previous studies reported that treatments with 10 or 100 nM of 1,25(OH)2D3 decrease the production of MCP-1, IL-6, and other pro-inflammatory cytokines and chemokines by pre-adipocytes and adipocytes from mice or humans [26,27,28,29]. The inhibition of IL-6 and TNFα production by human peripheral blood mononuclear cell(PBMC)s and bone marrow-derived mouse cells with in vitro 1,25(OH)2D3 treatment was observed, and the elevation of MAPK phosphatases 1, which would result in the suppression of the MAPK pathway, was suggested as a mechanism [30]. On the contrary, Sun et al. [31] reported that treatment with 10 nM of 1,25(OH)2D3 in 3T3-L1 adipocytes or RAW 264.7 macrophages upregulated the macrophage colony-stimulating factor (M-CSF), macrophage inflammatory protein (MIP), IL-6, and MCP-1 levels. Therefore, the effects of in vitro 1,25(OH)2D3 treatment on the production of inflammatory cytokines remain inconclusive. In addition, there are a limited number of studies that examined the effects of dietary vitamin D supplementation as well as 1,25(OH)2D3 treatment on immune cells from the adipose tissue of obese animals. Few in vivo studies have reported that dietary vitamin D could decrease IL-6 and MCP-1 in the adipose tissue of mice [26,28]; however, the anti-inflammatory effect of vitamin D was observed with the supplementation of 1,25(OH)2D3 (0.05 mg/kg diet), the biologically active form of vitamin D [26]. 

The aim of this study was to investigate whether vitamin D could alleviate inflammatory responses in adipose tissue, respectively, in adipocytes and SVCs. We evaluated whether dietary vitamin D supplementation would affect inflammatory responses and the subpopulation of immune cells in adipose tissue using high-fat diet-induced obese and lean mice. The effects of in vitro 1,25(OH)2D3 treatment on the production of pro-inflammatory cytokines and the expression of genes involved in inflammatory signaling pathways in the SVCs or adipocytes of obese and non-obese mice were investigated.

## 2. Materials and Methods 

### 2.1. Animals and Diets

Animals were housed in the specific pathogen free (SPF) animal facility at Seoul National University with an environmentally controlled temperature (23 ± 1 °C), relative humidity (50 ± 10%), and a 12-h light/12-h dark cycle. After 3 days of acclimation with the control diet, mice were randomly assigned to experimental groups. Food intake was measured four times per week and body weight was recorded weekly. All animal procedures were approved by the Institutional Animal Care and Use Committee of Seoul National University (approval numbers: SNU-141020-4, SNU-171010-1-1 and SNU-170404-10-2).

Three experiments (Exp.) were conducted, and five-week-old C57BL/6 male mice (Central Animal Laboratory) were used. In Exp. 1, mice were randomly assigned to 4 groups (*n* = 7~8 per each group) and fed experimental diets that differed in fat amount (10% or 45% kcal fat, CON or HFD) and vitamin D content (1000 or 25,000 IU vitamin D3/kg diet, DC or 25DS) ad libitum for 13 weeks (CON-DC, #103816; CON-25DS, #119321; HFD-DC, #103818; HFD-25DS, #119319; Dyets, Inc., Bethlehem, PA, USA). The composition of experimental diets is shown in Table 1. In Exp. 2, mice were divided into 4 groups (*n* = 8 per each group) and fed the diets that differed in fat amount (10% or 45% kcal fat: CON or HFD) and vitamin D3 content (1000 or 10,000 IU vitamin D3/kg of diet: DC or 10DS) ad libitum for 13 weeks (CON-DC, D12450H; CON-10DS, D17090501; HFD-DC, D12451; HFD-10DS, D17090502; Research Diets, New Brunswick, NJ, USA) (Supplementary Table S1). In Exp. 3, mice were fed the control (*n* = 9) or the high-fat diet (*n* = 10) (10% or 60% kcal fat: CON, #D12450B or HFD, #D12492, Research Diets) ad libitum for 12 weeks (Supplementary Table S2). Animals were fasted for 12 h and euthanized by CO_2_ asphyxiation. White adipose tissues (WAT) including perirenal, intraperitoneal, epididymal, and subcutaneous fats were collected and weighed. Visceral fats (perirenal, intraperitoneal, and epididymal fat) were placed in a dish containing sterile phosphate-buffered saline (PBS) with amphotericin (250 ng/mL) for stromal vascular cell isolation. 

### 2.2. Determination of Serum and Adipose Tissue 25(OH)D Levels

Serum 25(OH)D levels were measured by radioimmunoassay (RIA) using a commercial RIA kit (DiaSorin, Stillwater, MN, USA) according to the manufacturer’s instruction. The radioactivity was measured with an automatic gamma counter (2470 Wizard2, Perkin Elmer, Shelton, CT, USA). Adipose tissue 25(OH)D levels were measured by using a modification of the original method by Lipkie et al. [32]. A Shimadzu LCMS-8040 triple quadrupole mass spectrometer with a Shimadzu Nexara X ultra-high-performance liquid chromatography (UHPLC; Kyoto, Japan) system was used for the separation of 25(OH)D3 from adipose tissue extracts.

### 2.3. Adipocyte and Stromal Vascular Cell(SVC) Isolation

Visceral fats were cut into small pieces with scissors and put into DMEM (Grand Island, NY, USA) with 1 mg/mL collagenase type 2 (Sigma-Aldrich, St. Louis, MO, USA) and 2% bovine serum albumin (BSA) and were then incubated in a shaking 37 °C water bath (170 cycle/min) for 1 h. Tissue debris were removed using 200 μm nylon cell strainer. After centrifugation (1500 rpm, room temperature (RT), for 5 min), cells floating on the top were transferred to a new tube as adipocytes. The cell pellet at the bottom of the tube was washed with DMEM/10% FBS and resuspended with 1 mL of DMEM/10% FBS and 500 μL of 40% percoll in 10 × PBS. Reconstituted cells were slowly applied to a discontinuous percoll gradient, comprised of 40% and 70% percoll layers and were then centrifuged at 750× *g*, 20 °C for 20 min (excluding the acceleration and deceleration time) to acquire the immune cell fraction. The layer between the 40% and 70% percoll layers were transferred to a new tube as SVCs and centrifuged in RT at 1500 rpm for 5 min. Both adipocytes and SVCs were washed twice with DMEM/10% FBS and used for flow cytometry (FACS) analysis or cell culture. 

### 2.4. Flow Cytometric Analysis

For the analysis of SVCs subpopulation, SVCs were resuspended in a FACS-staining buffer (0.09% sodium azide, 1% FBS, 1× PBS based) and 2 × 10^5^ cells per sample were incubated with the antibodies at 4 °C for 30 mins. The antibodies used for analysis were purchased from BD Pharmingen (Franklin Lakes, NJ, USA), and specific cell surface markers are shown in Table 2. After staining, cells were washed and resuspended in a FACS-staining buffer with 4% formaldehyde, then analyzed using FACSCalibur II (BD Biosciences, SA, USA) and FlowJo software version 10 (Tree Star Inc., Ashland, OR, USA).

### 2.5. In Vitro 1,25(OH)2D3 Treatment

Adipocytes were cultured in a 6-well plate (4 × 10^6^ cells/well), and SVCs were cultured in a 12-well plate (2 × 10^5^ cells/well) with DMEM/10% FBS. To determine the effects of vitamin D, adipocytes and SVCs were incubated with either 1,25(OH)2D3 solution (10 nM, Sigma-Aldrich) in DMEM or 0.1% ethanol in DMEM (vehicle control) for 48 h and were stimulated with or without lipopolysaccharides (LPS ) (100 ng/mL, Sigma-Aldrich) during the last 24 h. Cells were incubated at 37 °C in 5% CO_2_ and a 100% humidified atmosphere. After 48 h of incubation, the supernatant was collected for cytokine analysis and cells (SVCs or adipocytes) were collected and stored at −80 °C for RNA or DNA isolation. 

### 2.6. Determination of Pro-Inflammatory Cytokines Production

MCP-1, IL-6, IL-1β, and TNF-α levels produced by adipocytes and SVCs were determined using Mouse ELISA MCP-1, IL-6, IL-1β, and TNF-α kits (BD Bioscience, San Diego, CA, USA), following the manufactures’ instructions. The absorbance was measured at 450 nm with a microplate spectrophotometer (Spectramax190, Molecular devices, CA, USA).

To normalize pro-inflammatory cytokine levels for the number of adipocytes, the total DNA from adipocytes was measured using a Quick-DNA™ Miniprep Plus kit (Zymo research, Irvine, CA, USA).

### 2.7. RNA Extraction from SVCs and Real-Time PCR

Total RNA was isolated from SVCs using RNAiso Plus (Takara bio., Shiga, Japan). The RNA quality was determined using agarose gel electrophoresis with the Gel Doc XR system (Bio-Rad Laboratories, Hercules, CA, USA). The total RNA (2 μg) sample was reverse transcribed into cDNA using PrimeScript™ 1st strand cDNA synthesis kit (Takara Bio Inc., Otsu, Shiga, Japan) with a thermal cycler (Applied Biosystems, Foster City, CA, USA). Each PCR reaction mixture contained synthesized cDNA, specific primers of a target gene, with SYBR Premix Ex Taq and ROX reference dye (Takara bio). mRNA levels of *Mcp-1*, regulated on the activation of a normal T cell expressed and secreted (*Rantes*), macrophage inflammatory protein-1 gamma (*Mip-1γ), Il-6, Il-1β, Tnf-α,* and interferon gamma (*Ifn-γ*) in the adipose tissue, *Tlr2*, *Tlr4*, dual specificity protein phosphatases (*Dusp)1, Dusp10*, nuclear factor of kappa light polypeptide gene enhanced in B-cells inhibitor alpha (*Iκbα*) in SVCs were determined with real-time quantitative PCR analysis using a StepOneTM Real-time PCR system (Applied Biosystems). The mRNA levels were normalized to the expression of the endogenous control gene (*Gapdh)* and all values are expressed as relative mRNA levels compared to the average level of the control group using the 2-ΔΔCT method. The oligonucleotide sequences of primers are presented in Table 3.

### 2.8. Statistical Analysis

Statistical analysis was performed using SPSS statistical software version 23 (IBM SPSS Statistics, Chicago, IL, USA). For Exp. 1 and 2, two-way ANOVA was used to evaluate the overall effects of the amount of dietary fat (CON or HFD) and dietary vitamin D content (DC or DS). Duncan’s multiple range post-hoc test was carried out when the effects of the amount of dietary fat and/or dietary vitamin D content were statistically significant. For Exp. 3, a Student’s *t*-test was conducted for comparing the effects of the amount of dietary fat between the CON and HFD group. In order to evaluate the effects of in vitro 1,25(OH)2D3 treatment on SVCs or adipocytes from the same tissue, a paired *t*-test was used. All data were represented as means ± SEMs, and *p* values less than 0.05 were considered statistically significant.

## 3. Results

### 3.1. Body Weight, Weight Change, WAT Weight, and Food Intake

There was no significant difference in body weight at week 0 among the groups. After 13 weeks, the HFD groups had higher body weight (*p* < 0.001) and WAT weight (*p* < 0.001) compared with the CON groups (Table 4, S3, and S4). There was no significant effect of dietary vitamin D supplementation (25,000 IU/kg diet) on WAT weight (Table 4). The average food intake (g/day) was not affected by dietary fat amount or vitamin D content. In addition, a lower dose of vitamin D supplementation (10,000 IU/kg diet) did not affect either body weight, WAT weight, or food intake (Table S3).

### 3.2. Serum and Epididymal Adipose Tissue 25(OH)D Levels

Serum 25(OH)D levels were significantly higher in the 25DS groups (25,000 IU/kg diet) when compared with the DC groups (Table 5). When vitamin D was supplemented at the level of 25,000 IU/kg diet, serum 25(OH)D levels were significantly lower in the HFD-25DS group compared with the CON-25DS group. These results were also confirmed with a lower dose of vitamin D supplementation (10,000 IU/kg diet) (Table S3).

Epididymal 25(OH)D levels of the 25DS groups were higher than those of the DC groups (3.3-fold, *p* < 0.001). The amount of dietary fat tended to have a significant effect on epididymal adipose tissue 25(OH)D levels (Table 5).

### 3.3. 1-Hydroxylase and Vdr Expression in Epididymal Adipose Tissue

Neither dietary fat amount nor dietary vitamin D content had a significant impact on the mRNA levels of *Cyp27b1* and *Vdr* (Figure S1).

### 3.4. Expression of Pro-Inflammatory Chemokines and Cytokines Expression in Epididymal Adipose Tissue

**Figure 1:**
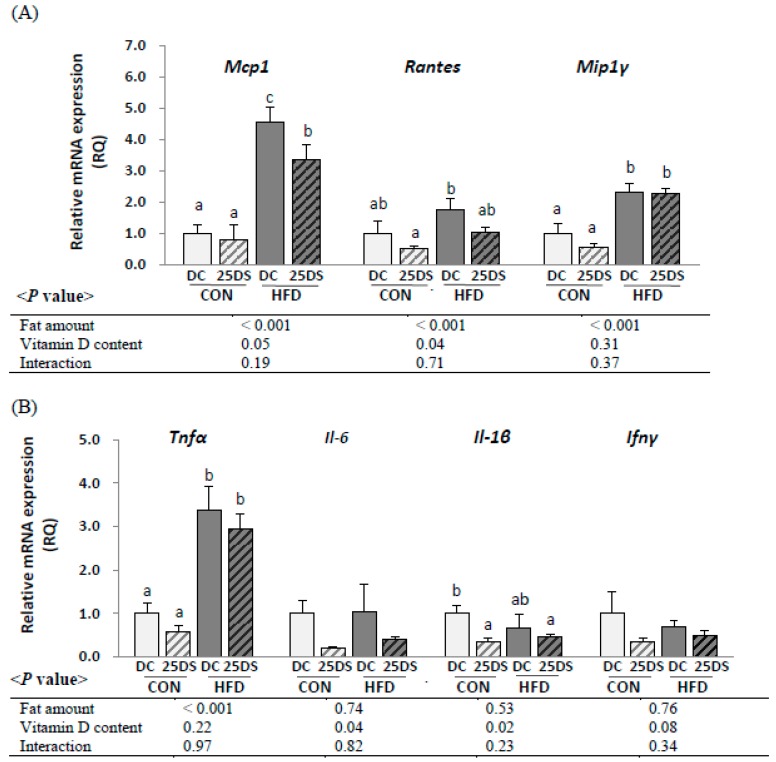
The mRNA levels of pro-inflammatory (**A**) chemokines and (**B**) cytokines in epididymal adipose tissue. Data are presented as mean ± SEM, *n* = 6~8 for each group. Two-way ANOVA was used to determine the significant effects of fat and vitamin D content and an interaction. ^ab^ Different superscripts indicate significant difference (*p* < 0.05) by Duncan’s multiple range test. CON: 10% kcal fat diet; HFD: 45% kcal fat diet; DC: 1000 IU vitamin D/kg diet; 25DS: 25,000 IU vitamin D/kg diet. *Mcp-1*, monocyte chemoattractant protein 1; *Rantes,* regulated on activation, normal T cell expressed and secreted; *Mip-1γ*, macrophage inflammatory protein-1 gamma; *Il-6*, interleukin 6; *Il-1β*, interleukin 1beta; *Tnf-α*, Tumor necrosis factor; *Ifn-γ*, interferon gamma.

To evaluate whether dietary vitamin D supplementation (25,000 IU/kg diet) could alleviate inflammatory responses in adipose tissue, the expression of pro-inflammatory cytokines was determined (Figure 1). The HFD groups had higher mRNA levels of epididymal *Mcp-1* (4.4-fold, *p* < 0.001), *Rantes* (1.9-fold, *p* < 0.001), *Mip-1γ* (2.9-fold, *p* < 0.001), and *Tnf α* (4.0-fold, *p* < 0.001) compared with the CON groups. Overall, vitamin D supplementation reduced the expression of *Mcp-1* (25% less, *p* = 0.05) and *Rantes* (43% less, *p* = 0.04). The HFD-25DS group had significantly lower *Mcp-1* mRNA levels compared with HFD-DC group (26% lower). The expression of *Mip-1γ* and *Tnf-α* was not significantly affected by vitamin D supplementation. Overall, the 25DS groups had lower mRNA levels of *Il-6* (70% less, *p* = 0.04) and *Il-1β* (52% less, *p* = 0.02) compared with the DC groups.

### 3.5. Subpopulation and the Number of Immune cells of SVCs in Visceral Adipose Tissue

**Figure 2:**
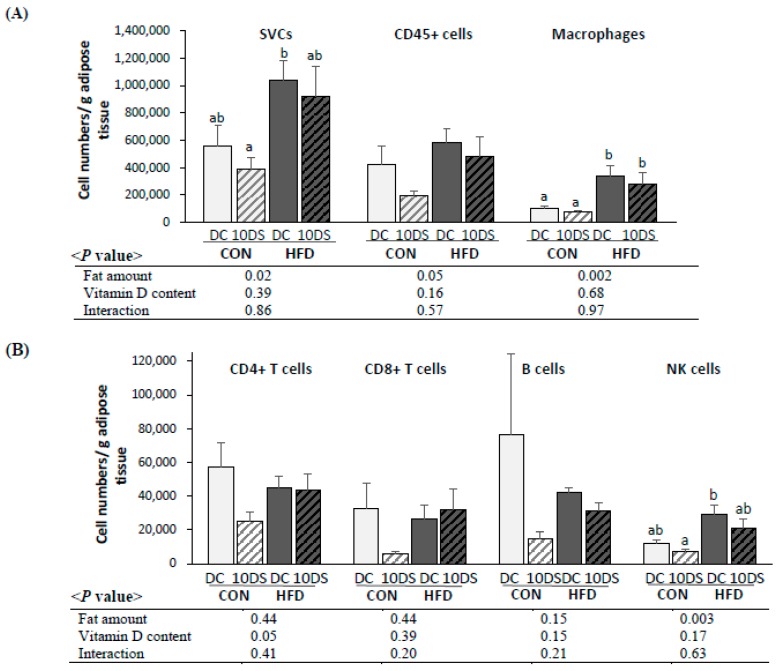
Cell numbers per gram of visceral adipose tissue. (**A**) Stromal vascular cell, CD45+ cell, macrophage, (**B**) CD4+ T cell, CD8+ T cell, B cell, and NK cell. Data are presented as mean ± SEM, *n* = 5~6 for each group. Two-way ANOVA was used to determine the significant effects of fat and vitamin D contents, and an interaction. ^ab^ Different superscripts indicate a significant difference (*p* < 0.05) by Duncan’s multiple range test. CON: 10% kcal fat diet; HFD: 45% kcal fat diet; DC: 1000 IU vitamin D/kg diet; 10DS: 10,000 IU vitamin D/kg diet.

The effects of a high-fat diet and vitamin D supplementation at a dose of 10,000 IU/kg on subpopulations of immune cells in SVCs were determined. The number of each type of immune cell per gram of visceral adipose tissue and total cell numbers are shown in Figure 2 and Table S5. The number of SVCs per g from visceral adipose tissue was significantly higher in the HFD groups than in the CON groups. Numbers of macrophages and NK cells (number/g tissue and total number) in SVCs were significantly higher in the HFD groups compared with the CON groups; however, there was no significant effect of vitamin D supplementation (Figure 2 and Figure S2).

Numbers of B cells, CD4+ T cells, and CD8+ T cells/g tissue were not different among groups (Figure 2), while the total number of CD4+ T cells was significantly higher in HFD groups than in CON groups (Table S5). The number of CD4+ T cells (numbers/g tissue and total numbers) tended to be lower in the 10DS groups compared with the DC groups (Figure 2: numbers/g, *p* = 0.05; Table S5: total number, *p* = 0.09).

### 3.6. Production of Pro-Inflammatory Cytokines by SVCs

**Figure 3:**
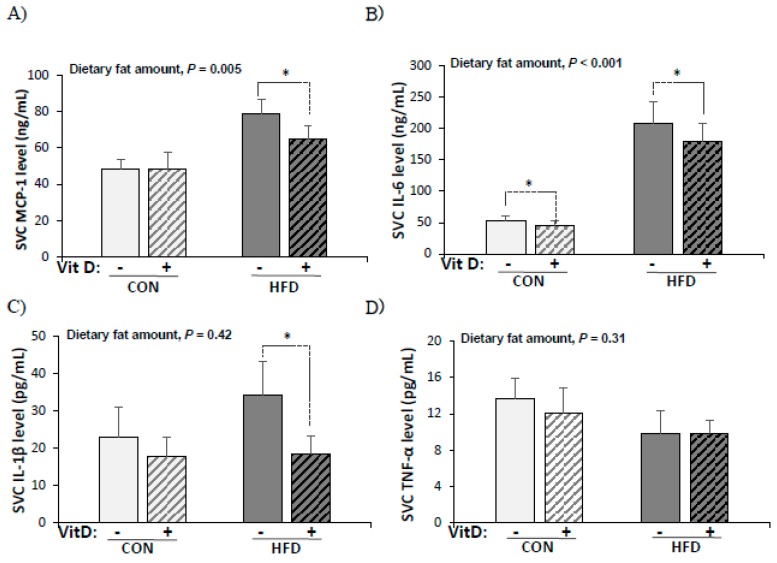
Production of pro-inflammatory cytokines by SVCs from CON and HFD mice (**A**) MCP-1 (ng/mL), (**B**) IL-6 (pg/mL), (**C**) IL-1β (pg/mL), and (**D**) TNF-α (pg/mL). SVCs were treated with vehicle (0.1% ethanol) or 1,25(OH)2D3 (10 nM) for 24 h before being stimulated with LPS (0.1 μg/mL) for another 24 hr. Data are presented as mean ± SEM, *n* = 9~10 for each group. A paired *t*-test was used to determine the significant effect of in vitro 1,25(OH)2D3 treatment and ^*^ indicates a significant difference (*p* < 0.05). A student’s *t* test was used to determine the significant effect of the amount of dietary fat. CON: 10% kcal fat diet; HFD: 60% kcal fat diet.

In order to elucidate whether in vitro 1,25(OH)2D3 treatment (10 nM) had a specific anti-inflammatory effect on SVCs from adipose tissue, levels of pro-inflammatory cytokines produced by SVCs from CON and HFD groups were determined (Figure 3). Significantly higher levels of MCP-1 (1.5-fold) and IL-6 (2.9-fold) were produced by LPS-stimulated SVCs from the HFD group compared with the CON group. MCP-1, IL-6, and IL-1β production from SVCs decreased significantly by in vitro 1,25(OH)2D3 treatment (MCP-1: 15% less, IL-6: 12% less, and IL-1β: 34% less) in the HFD group. In the CON group, only IL-6 production was decreased by in vitro 1,25(OH)2D3 treatment (16% less, *p* = 0.02 by paired *t*-test). The production of TNF-α by SVCs was not affected by either the high-fat diet or in vitro 1,25(OH)2D3 treatment.

### 3.7. Production of Pro-Inflammatory Cytokines by Adipocytes

**Figure 4:**
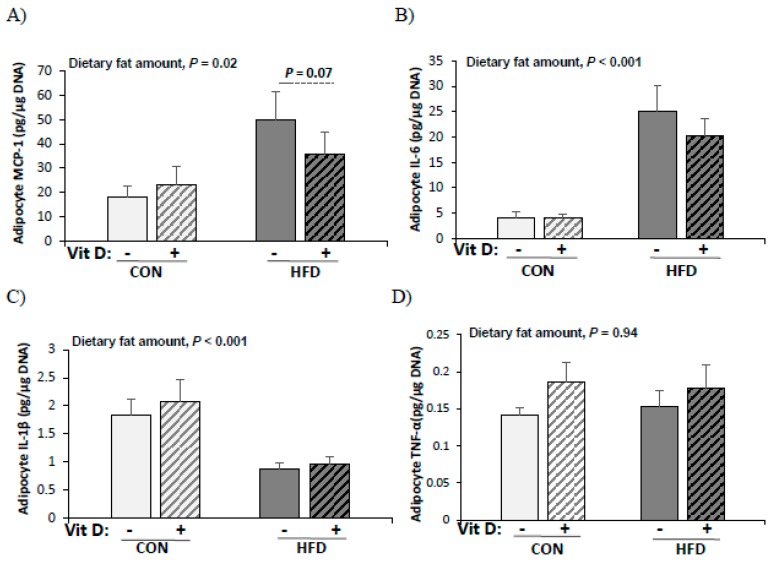
Production of pro-inflammatory cytokines by adipocytes from CON and HFD mice (**A**) MCP-1 (pg/μg DNA), (**B**) IL-6 (pg/μg DNA), (**C**) IL-1β (pg/μg DNA), and (**D**) TNF-α (pg/μg DNA). Adipocytes were treated with vehicle (0.1% ethanol) or 1,25(OH)2D3 (10 nM) for 24 h before being stimulated with LPS (0.1 μg/mL) for another 24 h. Production of each cytokine level was normalized with total cellular DNA. Data are presented as mean ± SEM, *n* = 9~10 for each group. A paired *t*-test was used to determine the significant effects of in vitro 1,25(OH)2D3 treatment and * indicates a significant difference (*p* < 0.05). A student’s *t* test was used to determine the significant effect of dietary fat amount. CON: 10% kcal fat diet; HFD: 60% kcal fat diet.

The effects of vitamin D on the production of pro-inflammatory cytokines by adipocytes were determined by in vitro 1,25(OH)2D3 treatment (10 nM). Adipocytes from the HFD group produced higher levels of MCP-1 (2.1-fold, *p* = 0.02) and IL-6 (5.5-fold, *p* < 0.001) than those from the CON group (Figure 4). However, the production of IL-1β was significantly higher in adipocytes from the CON group than those from the HFD group. There was a tendency of decreased MCP-1 production by adipocytes from the HFD group with in vitro 1,25(OH)2D3 treatment (28% less, *p* = 0.07 by paired *t*-test). The in vitro 1,25(OH)2D3 treatment had no significant effect on IL-6, IL-1β, and TNF-α production by adipocytes from the HFD group and MCP-1, IL-6, IL-1β, and TNF-α production by adipocytes from the CON group.

### 3.8. Expression of Genes Involved in Inflammatory Responses in SVCs

**Figure 5:**
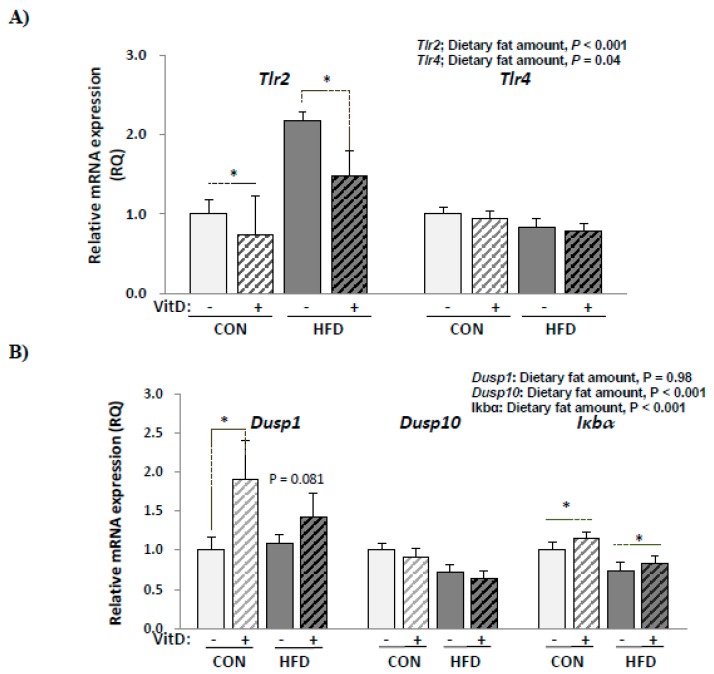
The mRNA levels of (**A**) *Tlr* and (**B**) *Dusp* and *Iκbα* in SVCs. Data are presented as mean ± SEM, *n* = 9~10 for each group. A paired *t*-test was used to determine the significant effects of in vitro 1,25(OH)2D3 treatment and * indicates a significant difference (*p* < 0.05). A student’s *t* test was used to determine the significant effects of the amount dietary fat. CON: 10% kcal fat diet; HFD: 60% kcal fat diet. *Tlr2*, toll like receptor 2; *Tlr4*, toll like receptor 4; *Dusp1*, dual specificity protein phosphatase 1; *Dusp10*, Dual specificity protein phosphatase 10; *Iκbα*, nuclear factor of kappa light polypeptide gene enhanced in B-cells inhibitor alpha.

The expression of genes involved in inflammatory signaling pathways was determined to see whether a decreased production of pro-inflammatory cytokines by 1,25(OH)2D3 treatment in SVCs could be attributed to the regulation of genes involved in MAPK and NF-κB signaling pathways (Figure 5). *Dusp1* and *Dusp10* are phosphatases that inactivate that MAP-kinase and *Iκbα* is an inhibitor of NF-κB transcription factor. *Tlr2* mRNA levels were higher in the HFD group compared with the CON group and significantly decreased by in vitro 1,25(OH)2D3 treatment. *Tlr2* expression decreased by 25% in the CON group and by 28% in the HFD group with in vitro 1,25(OH)2D3 treatment. However, in vitro 1,25(OH)2D3 treatment had no significant effect on *Tlr4* expression. *Dusp1* mRNA levels were not different between the CON and HFD groups. With 1,25(OH)2D3 treatment, *Dusp1* expression increased significantly in the CON group (44% higher, *p* = 0.01 by paired *t*-test) and tended to be increased in the HFD group (27% higher, *p* = 0.08 by paired *t*-test). *Dusp10* mRNA levels were significantly lower in the HFD group compared with the CON group. The in vitro 1,25(OH)2D3 treatment had no effect on *Dusp10* expression. *Iκbα* mRNA levels were 27.7% lower (*p* < 0.001) in the HFD group compared with the CON group and significantly increased by in vitro 1,25(OH)2D3 treatment in the CON (15.3% higher, *p* = 0.04 by paired t-test) and HFD groups (13.7% higher, *p* = 0.01 by paired *t*-test).

## 4. Discussion 

In this study, dietary vitamin D supplementation down-regulated the gene expression of pro-inflammatory cytokines in adipose tissue despite no difference in macrophage infiltration into the adipose tissue. The anti-inflammatory effects of vitamin D on adipose tissue might be due to the action of 1,25(OH)2D3 on inflammatory signaling in SVCs, since in vitro treatment of 1,25(OH)2D3 decreased pro-inflammatory cytokine production from SVCs possibly through regulating *Dusp1* and *Iκbα*.

Obesity has been known to increase the infiltration of immune cells into adipose tissue and the secretion of pro-inflammatory cytokines [11,38], which results in chronic adipose tissue inflammation. In the current study, the HFD groups had higher numbers of SVCs in adipose tissue (numbers/g tissue) compared with the CON groups, especially the numbers of macrophages and NK cells (per g tissue), which were 3 and 2 times higher, respectively, in the HFD groups. This is consistent with the reports that the numbers of macrophages as well as NK cells were higher in the obese mice that were fed 45% or 60% kcal fat for 12 weeks compared with lean mice [2,39]. Furthermore, SVCs and adipocytes from obese mice produced higher levels of MCP-1 and IL-6, respectively, than those from lean mice. These results confirm that HFD-induced obesity results in inflammatory responses in adipose tissue with increased immune cell numbers in adipose tissue and the up-regulated synthesis of inflammatory cytokines from both adipocytes and SVCs. 

In vitro 1,25(OH)2D3 treatment decreased MCP-1, IL-6, and IL-1β production by SVCs from obese mice (MCP-1: 15%, IL-6: 12%, and IL-1β: 34% decreased production); however, no significant difference was observed in the production of these cytokines by adipocytes with 1,25(OH)2D3 treatment. In obesity, the MCP-1 secreted by both hypertrophic adipocytes and M1 macrophages has been reported to accelerate macrophage infiltration into the adipose tissue and increase adipose tissue-derived IL-6 and IL-1β levels [11,40,41]. Elevated levels of IL-6 and IL-1β in adipose tissue have been reported to contribute to the risk of type 2 diabetes [42,43,44]. Although both adipocytes and non-adipocytes secrete these inflammatory cytokines, non-adipocytes (SVCs) produce 8~20 times more inflammatory cytokines, including MCP-1, IL-6, IL-1β, and TNF-α, compared with adipocytes, indicating that the majority of inflammatory cytokine released are by non-adipocytes [45]. Therefore, despite the lack of significant effects of 1,25(OH)2D on adipocytes, our results suggest that vitamin D could contribute to the alleviation of adipose tissue inflammation by reducing the production of inflammatory cytokines from SVCs. 

The effects of 1,25(OH)2D on adipose tissue inflammation have been investigated by other researchers [10,27,31,46,47]; however, contradictory results have been reported regarding its effects on adipocytes, and few studies have been conducted to examine the effect of vitamin D on SVCs. Sun et al. [31,48] reported that MCP-1 and IL-6 productions were increased by treatments with 10 nM of 1,25(OH)2D3 in 3T3-L1 cells [10,26] and differentiated human adipocytes [9], while others have shown that the production of IL-6, MCP-1, and IL-1β were reduced by 1,25(OH)2D3 in 3T3-L1 or differentiated human adipocyte; however, the cause of conflicting results were not delineated. Using 3T3-L1 and human adipocytes, Marcotorchino et al. [10] showed that vitamin D (100 nM of 1,25(OH)2D3) reduced IL-6, MCP-1, and IL-1B production and inactivated NF-κB by inducing IκBa. Gao et al. [27] reported the anti-inflammatory effect of vitamin D on SVCs by showing decreased MCP-1 and IL-6 levels with 10 or 100 nM of 1,25(OH)2D3 treatment on human pre-adipocytes. Although pre-adipocytes are one of the components of SVCs and have been shown to be involved in adipose tissue inflammation [49,50], major contributors to the adipose inflammation are immune cells, especially M1-like macrophages, which release a substantial amount of inflammatory cytokines [13]. In this study, 47%~50% of SVCs from HFD-induced obese mice were immune cells (CD45+ cells), of which more than 60% were macrophages. Since LPS can induce M1 polarization, the anti-inflammatory effects of vitamin D on SVCs in this study is likely to be due to its effect on immune cells in SVCs.

The activation of TLR signaling upon recognition of LPS or fatty acids released from lipolysis could induce pro-inflammatory cytokine production in adipose tissue through the NF-κB and MAPKs signaling pathways [14,20]. Increased levels of TLR2 protein have been reported in adipose tissues from obese or diabetic humans [23], and the expression of *Tlr2* mRNA was greater in HFD-induced obese mice [27]. In this study, *Tlr2* mRNA levels in SVCs were significantly higher (117% higher) in the HFD group compared with the CON group and were decreased by 1,25(OH)2D3 treatment in both the HFD and CON groups. TLR2 antisense-treated mice were insulin sensitive and resistant to chronic inflammation when fed a HFD for 8 weeks [51]. Therefore, down-regulated *Tlr2* expression by 1,25(OH)2D3 could have contributed to the alleviation of inflammatory signaling. In addition, both *Iκbα* and *Dusp1* mRNA levels were increased by 1,25(OH)2D3 treatment. *Iκbα* is the inhibitory subunit of NF-κB activation since it blocks the binding of NF-κB transcription factors to DNA [52] and Dusp1, which is known as the MAPK phosphatase 1 (MKP1) and plays a key role in the dephosphorylation and inactivation of MAPK in mammalian cells [53]; thus, elevated expression of *Iκbα* and *Dusp1* suggests that NF-κB and MAPK signaling pathways might be inhibited by 1,25(OH)2D3 treatment. Cohen-Lahav et al. [54] reported that 1,25(OH)2D3 treatment (100 nM) could reduce NF-κB activation by elevating *IκBα* levels in murine macrophage. Additionally, Zhang et al. [47] demonstrated that the anti-inflammatory effect of vitamin D in a bone marrow-derived macrophage is MKP1 dependent, by showing that mice lacking MKP-1 were unable to reduce inflammatory cytokines upon vitamin D treatment. Therefore, decreased *Tlr2* mRNA levels and inflammatory cytokine production by 1,25(OH)2D3 treatment in SVCs in this study might be partially due to up-regulated *Iκbα* and *Dusp1* levels. 

Overall, dietary vitamin D supplementation (25,000 IU/kg diet) resulted in lower mRNA levels of inflammatory cytokines (*Mcp-1, Rantes, Il-6*, and *Il-1β*) in adipose tissue by increasing adipose tissue 25(OH)D levels. However, the number of macrophages infiltrated into adipose tissue was not significantly changed by dietary vitamin D supplementation (10,000 IU vitamin D3/kg diet). Although the amount of dietary vitamin D supplemented was different between two experiments, the average serum 25(OH)D level in the 10,000 IU/kg diet-supplemented group (88.7 ng/mL) was not lower than its level in the 25,000 IU/kg diet-supplemented group (74.9 ng/mL); thus, even if vitamin D supplementation was at the 25,000 IU vitamin D3/kg diet level, it is likely that significant changes in immune cell numbers would not be observed. Dietary vitamin D might have reduced adipose tissue inflammation by regulating inflammatory gene expression directly without a significant reduction of macrophage infiltration; the anti-inflammatory effects of in vitro 1,25(OH)2D treatment on SVCs isolated from obese mice provides the mechanisms behind the in vivo effects of vitamin D.

## 5. Conclusions

In conclusion, dietary vitamin D supplementation attenuated inflammatory responses in adipose tissue from obese mice, and this might be attributed to the specific anti-inflammatory effect of 1,25(OH)2D3 in SVCs. Vitamin D supplementation did not result in reduced numbers of macrophages and NK cells in adipose tissue, but, 1,25(OH)2D3 treatment inhibited NF-κB and MAPK signaling in SVCs by increasing the expression of *Iκbα* and *Dusp1*. As a result, 1,25(OH)2D3 reduced the production of pro-inflammatory cytokines, which are increased by obesity. These findings suggest that obese people with chronic inflammation may benefit from vitamin D supplementation, however, clinical intervention studies to confirm these findings are needed. 

## Figures and Tables

**Table 1:** Composition of the experimental diets (Exp. 1) ^1^.

	CON (10% kcal fat)		HFD (45% kcal fat)	
	DC (1000 IU/kg of diet)	25DS (25,000 IU/kg of diet)	DC (1000 IU/kg of diet)	25DS (25,000 IU/kg of diet)
Casein (g)	200	200	200	200
L-Cystine (g)	3	3	3	3
Sucrose (g)	350	350	172.8	172.8
Cornstarch (g)	315	315	72.8	72.8
Dyetrose (g)	35	35	100	100
Soybean Oil (g)	45	45	45	45
t-BHQ (g)	0.009	0.009	0.009	0.009
Lard (g)	-	-	157.5	157.5
Cellulose (g)	50	50	50	50
Mineral Mix (g) ^2^	35	35	35	35
Vitamin Mix (g) (No vit D)	-	10	-	10
Vitamin Mix (g) ^3^	10	-	10	-
Vitamin D3 (400,000 IU/g)	-	0.0625	-	0.0625
Choline Bitartrate (g)	2	2	2	2
Total (g)	1045	1045	848.1	848.2
kcal/g diet	3.69	3.69	4.64	4.64

^1^ Resource: Dyets, Inc., Bethlehem, PA, USA. ^2^ 35 g of mineral mix (Dyets, #200000) provides 5.2 g calcium, 4 g phosphorus, 3.6 g potassium, 1 g sodium, 1.6 g chloride, 0.3 g sulfur, 0.5 g magnesium, 35 mg iron, 6 mg copper, 54 mg manganese, 30 mg zinc, 2 mg chromium, 0.2 mg iodine, 0.1 mg selenium, and 4.2 g sucrose. ^3^ A total of 10 g of vitamin mix (Dyets, #300050) provides 4000 IU vitamin A, 1000 IU vitamin D3, 50 IU vitamin E, 30 mg niacin, 16 mg pantothenic acid, 7 mg vitamin B6, 6 mg vitamin B1, 6 mg vitamin B2, 2 mg folic acid, 0.8 mg menadione, 0.2 mg biotin, 10 μg vitamin B12, and 9.8 g sucrose.

**Table 2:** Cell surface markers used for the flow cytometric analyses.

Cell	Antibodies	Company, Cat#	Clone
Macrophage	Percp-CD45	BD bioscience, 557235	30-F11
	FITC-CD11c	BD bioscience, 557400	HL3
	PE-F4/80	BD bioscience, 565410	T45–2342
	APC-CD11b	BD bioscience, 553312	M1/70
CD4 + _T cell	Percp-CD45	BD bioscience, 557235	30-F11
	APC-CD4	BD bioscience, 561091	RM4–5
CD8 + _T cell	Percp-CD45	BD bioscience, 557235	30-F11
	PE-CD8a	BD bioscience, 553033	53–6.7
B cell	Percp-CD45	BD bioscience, 557235	30-F11
	FITC-CD3	BD bioscience, 561798	17A2
	PE-CD19	BD bioscience, 553786	1D3
NK cell	Percp-CD45	BD bioscience, 557235	30-F11
	FITC-CD3	BD bioscience, 561798	17A2
	PE-NK1.1	BD bioscience, 553165	PK136
	APC-CD11b	BD bioscience, 553312	M1/70

**Table 3:** Primer sequences used in real-time PCR.

Gene ^1^	Forward Primer	Reverse Primer	Ref.^2^
*Cyp27b1*	GACGATGTTGGCTGTCTTCC	ATCTCTTCCCTTCGGCTTTG	[33]
*Vdr*	ATGTCCAGTGAGGGGGTGTA-	TGTCTGAGGAGCAACAGCAC	[33]
*Mcp1(Ccl2)*	AGGCATCACAGTCCGAGTCAC	CCTTTTCCACAACCACCTCAAG	[34]
*Rantes(Ccl5)*	CTTGAACCCACTTCTTCTCTGG	TGCTGCTTTGCCTACCTCTC	[35]
*Mip-1γ(Ccl9)*	TGGGTGTTATGTAGTCAAAGGAG	GAGGAAGGAGAGGGCAGTATG	-
*Il-6*	CATTTCCACGATTTCCCAGAGA	TCCATCCAGTTGCCTTCTTGGG	[34]
*Il-1β*	GCAACTGTTCCTGAACTCAACT	ATCTTTTGGGGTCCGTCAACT	[34]
*Tnf-α*	CTGGAAAGGTCTGAAGGTAGGAAGG	AACACAAGATGCTGGGACAGTGA	[34]
*Ifn- γ*	TGGACCTGTGGGTTGTTGAC	GAACTGGCAAAAGGATGGTG	[35]
*Tlr2*	CTTCATCTACGGGCAGTGGT	TTTGCTGGGCTGACTTCTCT	-
*Tlr4*	TTTCACCTCTGCCTTCACTACA	GGGACTTCTCAACCTTCTCAA	[36]
*Dusp1*	CGGTGAAGCCAGATTAGGAG	AGCGAAGAAGGAGCGACAA	[37]
*Dusp10*	AGGAAAGAAGAGCGACAAGC	TCAAAGGCAAACGACCAAT	-
*Iκbα*	CAGCATCTCCACTCCGTCCT	ACATCAGCCCCACATTTCA	-
*Gapdh*	GGAGAAACCTGCCAAGTA	AAGAGTGGGAGTTGCTGTTG	[33]

^1^*Cyp27b1*, cytochrome P450 27B1; *Vdr,* vitamin D receptor; *Mcp-1*, monocyte chemoattractant protein 1; *Rantes*, regulated on activation, normal T cell expressed and secreted; *Mip-1γ*, macrophage inflammatory protein-1 gamma; *IL-6,* interleukin 6; *IL-1β,* interleukin 1beta; *Tnf-α*, tumor necrosis factor; *Ifn- γ,* interferon gamma; *Tlr2,* toll like receptor 2; *Tlr4*, toll like receptor 4; *Dusp1*, dual specificity protein phosphatase 1; *Dusp10*, Dual specificity protein phosphatase 10; *Iκbα*, nuclear factor of kappa light polypeptide gene enhanced in B-cells inhibitor alpha *, Gapdh*, glyceraldehyde 3-phosphate dehydrogenase. ^2^ Specificity for each designed primer was confirmed using melt curve analysis and Primer-BLAST in National Center for Biotechnology Information (NCBI).

**Table 4:** Body weight, weight gain, body fat, and food intake of mice in the CON-DC, CON-25DS, HFD-DC, and HFD-25DS groups ^1,2^.

	CON		HFD		*p*-Value		
	DC (*n* = 8)	25DS (*n* = 7)	DC (*n* = 7)	25DS (*n* = 7)	Fat Amount	Vitamin D Content	Interaction
Body weight at 0 week (g)	22.8 ± 0.4	23.2 ± 0.3	22.6 ± 0.3	22.8 ± 0.3	0.32	0.38	0.65
Body weight at 13 week (g)	34.9 ± 0.5 ^a^	34.5 ± 1.2 ^a^	49.3 ± 1.3 ^c^	46.4 ± 0.9 ^b^	<0.001	0.12	0.22
Weight gain (g)	12.1 ± 0.8 ^a^	11.3 ± 1.3 ^a^	26.6 ± 1.3 ^c^	23.6 ± 1.0 ^b^	<0.001	0.11	0.33
WAT weight ^3^ (g)	2.70 ±0.27 ^a^	2.62 ±0.33 ^a^	5.92 ± 0.17 ^b^	5.68 ± 0.24 ^b^	<0.001	0.55	0.76
Average food intake (g/day)	2.90 ± 0.07	3.00 ± 0.05	3.17 ± 0.17	2.80 ± 0.05	0.73	0.17	0.02

^1^ Two-way ANOVA was used to determine the significant effects of fat and vitamin D content and an interaction. ^ab^ Different superscripts indicate significant difference (*p* < 0.05) by Duncan’s multiple range test. Data are presented as mean ± SEM. ^2^ CON: 10% kcal fat; HFD: 45% kcal fat diet; DC: 1000 IU vitamin D/kg diet; 25DS: 25,000 IU vitamin D/kg diet. ^3^ WAT: White adipose tissue weight included epididymal, subcutaneous, retroperitoneum, and perinephric fat.

**Table 5:** Serum and epididymal adipose tissue 25(OH)D levels ^1,2^.

	CON		HFD		*p*-Value		
	DC	25DS	DC	25DS	Fat Amount	Vitamin D Content	Interaction
Serum 25(OH)D levels (ng/mL)	24.0 ± 2.5 ^a^	83.1 ± 1.6 ^c^	29.4 ± 1.7 ^a^	66.7 ± 3.7 ^b^	0.04	<0.001	<0.001
Adipose 25(OH)D levels (ng/g tissue)	3.4 ± 0.4 ^a^	18.1 ± 2.2 ^b^	8.0 ± 1.5 ^a^	19.5 ± 2.1 ^b^	0.09	<0.001	0.36

^1^ Two-way ANOVA was used to determine the significant effects of fat and vitamin D contents and an interaction. ^a,b^ Different superscripts indicate significant difference (*p* < 0.05) by Duncan’s multiple range test. Data are presented as mean ± SEM. *n* = 6~7 for each group. ^2^ CON: 10% kcal fat; HFD: 45% kcal fat diet; DC: 1000 IU vitamin D/kg diet; 25DS: 25,000 IU vitamin D/kg diet.

## Supplementary Materials

The following are available online at https://www.mdpi.com/2072-6643/12/2/364/s1, Table S1: Composition of the experimental diets (Exp. 2), Table S2: Composition of the experimental diets (Exp. 3), Table S3. Body weight, weight gain, body fat, food intake, and serum 25(OH)D level of mice in the CON-DC, CON-10DS, HFD-DC and HFD-10DS groups, Table S4. Body weight, weight gain, body fat, and food intake of mice in the CON and HFD groups (Exp. 3), Table S5. Visceral fat tissue weights (g) and total cell numbers of Stromal vascular cell(SVC)s, CD45+ cells, macrophages, NK cells, B cells, CD4+ T cells, and CD8+ T cells in visceral adipose tissue, Figure S1: The mRNA levels of *Cyp27b1* and *Vdr* in epididymal adipose tissue, Figure S2: Flow cytometric dot plots of macrophage from visceral adipose tissue

## References

[1] Fantuzzi G. (2005). Adipose tissue, adipokines, and inflammation. J. Allergy Clin. Immunol..

[2] Weisberg S.P., McCann D., Desai M., Rosenbaum M., Leibel R.L., Ferrante A.W. (2003). Obesity is associated with macrophage accumulation in adipose tissue. J. Clin. Investig..

[3] Liu Q., Bengmark S., Qu S. (2010). The role of hepatic fat accumulation in pathogenesis of non-alcoholic fatty liver disease (NAFLD). Lipids Health Dis..

[4] Stojsavljević S., Gomerčić Palčić M., Virović Jukić L., Smirčić Duvnjak L., Duvnjak M. (2014). Adipokines and proinflammatory cytokines, the key mediators in the pathogenesis of nonalcoholic fatty liver disease. J. Gastroenterol..

[5] Pereira-Santos M., Costa P.R., Assis A.M., Santos C.A., Santos D.B. (2015). Obesity and vitamin D deficiency: A systematic review and meta-analysis. Obesity reviews: An official journal of the International. Pediatr. Obes..

[6] Mezza T., Muscogiuri G., Sorice G.P., Prioletta A., Salomone E., Pontecorvi A., Giaccari A. (2012). Vitamin D deficiency: A new risk factor for type 2 diabetes?. Ann. Nutr. Metab..

[7] Cheng S., Massaro J.M., Fox C.S., Larson M.G., Keyes M.J., McCabe E.L., Robins S.J., O’Donnell C.J., Hoffmann U., Jacques P.F. (2010). Adiposity, cardiometabolic risk, and vitamin D status: The Framingham Heart Study. Diabetes.

[8] Roth C.L., Elfers C.T., Figlewicz D.P., Melhorn S.J., Morton G.J., Hoofnagle A., Yeh M.M., Nelson J.E., Kowdley K.V. (2012). Vitamin D deficiency in obese rats exacerbates nonalcoholic fatty liver disease and increases hepatic resistin and Toll-like receptor activation. Hepatol. (Baltimore, Md.).

[9] Ding C., Wilding J.P., Bing C. (2013). 1,25-dihydroxyvitamin D3 protects against macrophage-induced activation of NFkappaB and MAPK signalling and chemokine release in human adipocytes. PLoS ONE.

[10] Marcotorchino J., Gouranton E., Romier B., Tourniaire F., Astier J., Malezet C., Amiot M.J., Landrier J.F. (2012). Vitamin D reduces the inflammatory response and restores glucose uptake in adipocytes. Mol. Nutr. Food Res..

[11] Tilg H., Moschen A.R. (2006). Adipocytokines: Mediators linking adipose tissue, inflammation and immunity. Nat. Rev. Immunol..

[12] Lehr S., Hartwig S., Lamers D., Famulla S., Muller S., Hanisch F.G., Cuvelier C., Ruige J., Eckardt K., Ouwens D.M. (2012). Identification and validation of novel adipokines released from primary human adipocytes. Mol. Cell. Proteom. MCP.

[13] Choe S.S., Huh J.Y., Hwang I.J., Kim J.I., Kim J.B. (2016). Adipose Tissue Remodeling: Its Role in Energy Metabolism and Metabolic Disorders. Front. Endocrinol..

[14] Watanabe Y., Nagai Y., Takatsu K. (2013). Activation and regulation of the pattern recognition receptors in obesity-induced adipose tissue inflammation and insulin resistance. Nutrients.

[15] Nishimura S., Manabe I., Nagasaki M., Eto K., Yamashita H., Ohsugi M., Otsu M., Hara K., Ueki K., Sugiura S. (2009). CD8+ effector T cells contribute to macrophage recruitment and adipose tissue inflammation in obesity. Nat. Med..

[16] Feuerer M., Herrero L., Cipolletta D., Naaz A., Wong J., Nayer A., Lee J., Goldfine A.B., Benoist C., Shoelson S. (2009). Lean, but not obese, fat is enriched for a unique population of regulatory T cells that affect metabolic parameters. Nat. Med..

[17] Fabbrini E., Cella M., McCartney S.A., Fuchs A., Abumrad N.A., Pietka T.A., Chen Z., Finck B.N., Han D.H., Magkos F. (2013). Association between specific adipose tissue CD4+ T-cell populations and insulin resistance in obese individuals. Gastroenterology.

[18] Jagannathan-Bogdan M., McDonnell M.E., Shin H., Rehman Q., Hasturk H., Apovian C.M., Nikolajczyk B.S. (2011). Elevated proinflammatory cytokine production by a skewed T cell compartment requires monocytes and promotes inflammation in type 2 diabetes. J. Immunol. (Baltimore, Md.: 1950).

[19] Del Valle H.B., Yaktine A.L., Taylor C.L., Ross A.C. (2011). Dietary Reference Intakes for Calcium and Vitamin D.

[20] Uematsu S., Akira S. (2006). Toll-like receptors and innate immunity. J. Mol. Med. (Berlin, Germany).

[21] Lee J.Y., Sohn K.H., Rhee S.H., Hwang D. (2001). Saturated fatty acids, but not unsaturated fatty acids, induce the expression of cyclooxygenase-2 mediated through Toll-like receptor 4. J. Biol. Chem..

[22] Wong S.W., Kwon M.J., Choi A.M., Kim H.P., Nakahira K., Hwang D.H. (2009). Fatty acids modulate Toll-like receptor 4 activation through regulation of receptor dimerization and recruitment into lipid rafts in a reactive oxygen species-dependent manner. J. Biol. Chem..

[23] Creely S.J., McTernan P.G., Kusminski C.M., Fisher f M., Da Silva N.F., Khanolkar M., Evans M., Harte A.L., Kumar S. (2007). Lipopolysaccharide activates an innate immune system response in human adipose tissue in obesity and type 2 diabetes. Am. J. Physiol. Endocrinol. Metab..

[24] Shi H., Kokoeva M.V., Inouye K., Tzameli I., Yin H., Flier J.S. (2006). TLR4 links innate immunity and fatty acid-induced insulin resistance. J. Clin. Investig..

[25] Mutt S.J., Hypponen E., Saarnio J., Jarvelin M.R., Herzig K.H. (2014). Vitamin D and adipose tissue-more than storage. Front. Physiol..

[26] Lira F.S., Rosa J.C., Cunha C.A., Ribeiro E.B., do Nascimento C.O., Oyama L.M., Mota J.F. (2011). Supplementing alpha-tocopherol (vitamin E) and vitamin D3 in high fat diet decrease IL-6 production in murine epididymal adipose tissue and 3T3-L1 adipocytes following LPS stimulation. Lipids Health Dis..

[27] Gao D., Trayhurn P., Bing C. (2013). 1,25-Dihydroxyvitamin D3 inhibits the cytokine-induced secretion of MCP-1 and reduces monocyte recruitment by human preadipocytes. Int. J. Obes. (2005).

[28] Karkeni E., Marcotorchino J., Tourniaire F., Astier J., Peiretti F., Darmon P., Landrier J.F. (2015). Vitamin D limits chemokine expression in adipocytes and macrophage migration in vitro and in male mice. Endocrinology.

[29] Lorente-Cebrian S., Eriksson A., Dunlop T., Mejhert N., Dahlman I., Astrom G., Sjolin E., Wahlen K., Carlberg C., Laurencikiene J. (2012). Differential effects of 1alpha,25-dihydroxycholecalciferol on MCP-1 and adiponectin production in human white adipocytes. Eur. J. Nutr..

[30] Zhang Y., Leung D.Y., Richers B.N., Liu Y., Remigio L.K., Riches D.W., Goleva E. (2012). Vitamin D inhibits monocyte/macrophage proinflammatory cytokine production by targeting MAPK phosphatase-1. J. Immunol. (Baltimore, Md.: 1950).

[31] Sun X., Zemel M.B. (2008). Calcitriol and calcium regulate cytokine production and adipocyte-macrophage cross-talk. J. Nutr. Biochem..

[32] Lipkie T.E., Janasch A., Cooper B.R., Hohman E.E., Weaver C.M., Ferruzzi M.G. (2013). Quantification of vitamin D and 25-hydroxyvitamin D in soft tissues by liquid chromatography-tandem mass spectrometry. J. Chromatogr. B Analyt. Technol. Biomed. Life Sci..

[33] Jung Y.S., Wu D., Smith D., Meydani S.N., Han S.N. (2018). Dysregulated 1,25-dihydroxyvitamin D levels in high-fat diet-induced obesity can be restored by changing to a lower-fat diet in mice. Nutr. Res..

[34] Park C.Y., Park S., Kim M.S., Kim H.K., Han S.N. (2017). Effects of mild calorie restriction on lipid metabolism and inflammation in liver and adipose tissue. Biochem. Biophys. Res. Commun..

[35] Lee G.Y., Park C.Y., Cha K.S., Lee S.E., Pae M., Han S.N. (2018). Differential effect of dietary vitamin D supplementation on natural killer cell activity in lean and obese mice. J. Nutr. Biochem..

[36] Li M., Song L., Gao X., Chang W., Qin X. (2012). Toll-like receptor 4 on islet beta cells senses expression changes in high-mobility group box 1 and contributes to the initiation of type 1 diabetes. Exp. Mol. Med..

[37] Dias J.D., Rito T., Torlai Triglia E., Kukalev A., Ferrai C., Chotalia M., Brookes E., Kimura H., Pombo A. (2015). Methylation of RNA polymerase II non-consensus Lysine residues marks early transcription in mammalian cells. eLife.

[38] Castoldi A., Naffah de Souza C., Câmara N.O., Moraes-Vieira P.M. (2015). The Macrophage Switch in Obesity Development. Front. Immunol..

[39] Lee B.C., Kim M.S., Pae M., Yamamoto Y., Eberle D., Shimada T., Kamei N., Park H.S., Sasorith S., Woo J.R. (2016). Adipose Natural Killer Cells Regulate Adipose Tissue Macrophages to Promote Insulin Resistance in Obesity. Cell Metab..

[40] Makki K., Froguel P., Wolowczuk I. (2013). Adipose tissue in obesity-related inflammation and insulin resistance: Cells, cytokines, and chemokines. ISRN Inflamm..

[41] Trayhurn P., Wood I.S. (2004). Adipokines: Inflammation and the pleiotropic role of white adipose tissue. Br. J. Nutr..

[42] Gao D., Madi M., Ding C., Fok M., Steele T., Ford C., Hunter L., Bing C. (2014). Interleukin-1β mediates macrophage-induced impairment of insulin signaling in human primary adipocytes. Am. J. Physiol. Endocrinol. Metab..

[43] Febbraio M.A. (2014). Role of interleukins in obesity: Implications for metabolic disease. Trends Endocrinol. Metab. TEM.

[44] Satoh T., Otsuka A., Contassot E., French L.E. (2015). The inflammasome and IL-1beta: Implications for the treatment of inflammatory diseases. Immunotherapy.

[45] Fain J.N. (2010). Release of inflammatory mediators by human adipose tissue is enhanced in obesity and primarily by the nonfat cells: A review. Mediat. Inflamm..

[46] Marcotorchino J., Tourniaire F., Astier J., Karkeni E., Canault M., Amiot M.J., Bendahan D., Bernard M., Martin J.C., Giannesini B. (2014). Vitamin D protects against diet-induced obesity by enhancing fatty acid oxidation. J. Nutr. Biochem..

[47] Zhang X.L., Guo Y.F., Song Z.X., Zhou M. (2014). Vitamin D prevents podocyte injury via regulation of macrophage M1/M2 phenotype in diabetic nephropathy rats. Endocrinology.

[48] Sun X., Morris K.L., Zemel M.B. (2008). Role of calcitriol and cortisol on human adipocyte proliferation and oxidative and inflammatory stress: A microarray study. J. Nutr..

[49] Caer C., Rouault C., Le Roy T., Poitou C., Aron-Wisnewsky J., Torcivia A., Bichet J.C., Clement K., Guerre-Millo M., Andre S. (2017). Immune cell-derived cytokines contribute to obesity-related inflammation, fibrogenesis and metabolic deregulation in human adipose tissue. Sci. Rep..

[50] Sorisky A., Molgat A.S., Gagnon A. (2013). Macrophage-induced adipose tissue dysfunction and the preadipocyte: Should I stay (and differentiate) or should I go?. Adv. Nutr. (Bethesda, Md.).

[51] Caricilli A.M., Nascimento P.H., Pauli J.R., Tsukumo D.M., Velloso L.A., Carvalheira J.B., Saad M.J. (2008). Inhibition of toll-like receptor 2 expression improves insulin sensitivity and signaling in muscle and white adipose tissue of mice fed a high-fat diet. J. Endocrinol..

[52] Gupta S.C., Sundaram C., Reuter S., Aggarwal B.B. (2010). Inhibiting NF-κB activation by small molecules as a therapeutic strategy. Biochim. Biophys..

[53] Owens D.M., Keyse S.M. (2007). Differential regulation of MAP kinase signalling by dual-specificity protein phosphatases. Oncogene.

[54] Cohen-Lahav M., Shany S., Tobvin D., Chaimovitz C., Douvdevani A. (2006). Vitamin D decreases NFkappaB activity by increasing IkappaBalpha levels. Nephrology, dialysis, transplantation: Official publication of the European Dialysis and Transplant Association. Eur. Ren. Assoc..

