# Supplementary material for: Effects of 1,25-Dihydroxyvitamin D3 on the Inflammatory Responses of Stromal Vascular Cells and Adipocytes from Lean and Obese Mice
Source: Nutrients. 2020 Jan 30;12(2):364. doi: 10.3390/nu12020364 (PMC7071143; doi:10.3390/nu12020364)
Supplement: Supplementary file 1 [file nutrients-12-00364-s001.pdf]

Supplementary Files

Table S1. Composition of the experimental diets (2)

	**CON (10% kcal fat)**		**HFD (45% kcal fat)**	
	**DC (1,000 IU/kg of diet)**	**10DS (10,000 IU/kg of diet)**	**DC (1,000 IU/kg of diet)**	**10DS (10,000 IU/kg of diet)**
Casein, 30 Mesh	200	200	200	200
L-Cystine	3	3	3	3
Corn Starch	452.2	452.2	72.8	72.8
Maltodextrin 10	75	75	100	100
Sucrose	172.8	172.8	172.8	172.8
Cellulose, BW200	50	50	50	50
Soybean Oil	25	25	25	25
Lard	20	20	177.5	177.5
Mineral Mix [1]	10	10	10	10
DiCalcium Phosphate	13	13	13	13
Calcium Carbonate	5.5	5.5	5.5	5.5
Potassium Citrate, 1 H_2O	16.5	16.5	16.5	16.5
Vitamin Mix [2]	10	0	10	0
Vitamin Mix (No vitD)	0	10	0	10
Vitamin D3 (100,000IU/g)	0	0.09	0	0.09
Choline Bitartrate	2	2	2	2
FD&C Yellow Dye #5	0.04	0	0	0.025
FD&C Red Dye #40	0.01	0	0.05	0
FD&C Blue Dye #1		0.05	0	0.025
Total	1,055.05	1,055.14	858.15	858.24
kcal/g diet	3.85	3.84	4.73	4.73

Resource: Research Diets Inc., New Brunswick, NJ, USA

[1] 10 g of Mineral Mix (Research Diets Inc., #S10026) provides 1.0 g of Na, 1.6 g of Cl, 0.5 g of Mg, 0.33 g of S, 59 mg of Mn, 37 mg of Fe, 29 mg of Zn, 6.0 mg of Cu, 2.0 mg of Cr, 1.6 mg of Mo, 0.16 mg of Se, 0.9 mg of Fl, 0.2 mg of I, and 3.99 g of sucrose.

[2] 10 g of Vitamin Mix (Research Diets Inc., #V10001) provides 4000 IU of vitamin A, 1000 IU of vitamin D_3, 50 IU of vitamin E, 0.5 mg of menadione, 0.2 mg of biotin, 10 μg of vitamin B_{12}, 2 mg of folic acid, 30 mg of niacin, 16 mg of pantothenic acid, 7 mg of vitamin B_6, 6 mg of vitamin B_2, 6 mg of vitamin B_1, and 9.78 g of sucrose.

Table S2. Composition of the experimental diets (3)

	Control diet (g), CON (10% kcal Fat)	High fat diet (g), HFD (60% kcal Fat)
Casein, 30 Mesh	200	200
L-Cystine	3	3
Corn Starch	315	72.8
Maltodextrin 10	35	125
Sucrose	350	68.8
Cellulose, BW200	50	50
Soybean Oil	25	25
Lard	20	245
Mineral Mix [1]	10	10
DiCalcium Phosphate	13	13
Calcium Carbonate	5.5	5.5
Potassium Citrate, 1 H_2O	16.5	16.5
Vitamin Mix [2]	10	10
Choline Bitartrate	2	2
FD&C Yellow Dye #5	0.05	0
FD&C Red Dye #40	0	0.05
Total	1,055.05	773.85
kcal/g diet	3.85	5.24

Resource: Research Diets Inc., New Brunswick, NJ, USA

[1] 10 g of Mineral Mix (Research Diets Inc., #S10026) provides 1.0 g of Na, 1.6 g of Cl, 0.5 g of Mg, 0.33 g of S, 59 mg of Mn, 37 mg of Fe, 29 mg of Zn, 6.0 mg of Cu, 2.0 mg of Cr, 1.6 mg of Mo, 0.16 mg of Se, 0.9 mg of Fl, 0.2 mg of I, and 3.99 g of sucrose.

[2] 10 g of Vitamin Mix (Research Diets Inc., #V10001) provides 4000 IU of vitamin A, 1000 IU of vitamin D_3, 50 IU of vitamin E, 0.5 mg of menadione, 0.2 mg of biotin, 10 µg of vitamin B_{12}, 2 mg of folic acid, 30 mg of niacin, 16 mg of pantothenic acid, 7 mg of vitamin B_6, 6 mg of vitamin B_2, 6 mg of vitamin B_1, and 9.78 g of sucrose.

Table S3. Body weight, weight gain, body fat, food intake, and serum 25(OH)D level of mice in the CON-DC, CON-10DS, HFD-DC and HFD-10DS groups[1,2]

	CON		HFD		*P*-value		
	**DC (n=8)**	**10DS (n=8)**	**DC (n=8)**	**10DS (n=8)**	**Fat amount**	**Vitamin D content**	**Interaction**
Body wt. at 0 week (g)	18.6 ± 1.5	18.8 ± 1.2	18.6 ± 1.2	18.5 ± 0.9	0.80	0.94	0.65
Body wt. at 13 week (g)	30.1 ± 2.0[a]	29.2 ± 2.6[a]	40.2 ± 4.0[b]	40.7 ± 3.5[b]	< 0.001	0.84	0.57
Weight gain (g)	11.5 ± 1.5[a]	10.4 ± 2.4[a]	22.0 ± 2.8[b]	22.5 ± 3.3[b]	< 0.001	0.73	0.38
White adipose tissue wt.[3] (g)	1.89 ± 0.46[a]	1.76 ± 0.60[a]	5.21± 0.80[b]	5.28 ± 0.77[b]	< 0.001	0.91	0.68
Average food intake (g/day)	2.74 ± 0.11[c]	2.65 ± 0.06[b]	2.54 ± 0.04[a]	2.65 ± 0.07[b]	0.001	0.56	0.001
Serum 25(OH)D (ng/mL)	37.1± 4.8[a]	93.9 ± 8.2[c]	34.5 ± 3.2[a]	83.4 ± 6.6[b]	0.005	< 0.001	0.07

[1]Two-way ANOVA was used to determine the significant effects of fat and vitamin D contents, and an interaction. [ab]Different superscripts indicate significant difference ($P < 0.05$) by Duncan's multiple range test. The data are presented as means ± SEM.
[2]CON: 10% kcal fat diet; HFD: 45% kcal fat diet; DC: 1,000 IU vitamin D/kg diet; 10DS: 10,000 IU vitamin D/kg diet.
[3]White adipose tissue weight included epididymal, subcutaneous, retroperitoneum, and perinephric fat.

Table S4. Body weight, weight gain, body fat, and food intake of mice in the CON and HFD groups

	CON (n = 9)	HFD (n = 10)	*P* value [2]
Body weight at 0 week (g)	19.8 ± 0.3	19.9 ± 1.1	0.89
Body weight at 11 week (g)	30.9 ± 0.6	42.7 ± 1.2	< 0.001
WAT weight [2] (g)	2.48 ± 0.12	5.55 ± 0.21	< 0.001
Average food intake (g/day)	2.68 ± 0.04	2.33 ± 0.04	< 0.001
Average energy intake (kcal/day)	10.3 ± 0.2	12.2 ± 0.2	< 0.001

[1] The data are presented as means ± SEM, Student's *t* test was used to determine the significant effect of obesity ($P < 0.05$). CON: 10% kcal fat diet; HFD: 60% kcal fat diet.

[2] WAT includes perirenal, intraperitoneal, epididymal, and subcutaneous fat.

Table S5. Visceral fat tissue weight (g) and total cell numbers of stromal vascular cell(SVC)s, CD45+ cells, macrophages, NK cells, B cells, CD4+ T cells, and CD8 T cells in visceral adipose tissue[1,2]

	CON		HFD		*P*-value		
	**DC (n=5)**	**10DS (n=5)**	**DC (n=6)**	**10DS (n=6)**	**Fat amount**	**VitaminD content**	**Interaction**
Visceral fat weights[3] (g)	0.97 ± 0.09[a]	0.86 ± 0.15[a]	2.02 ± 0.01[b]	2.01 ± 0.01[b]	< 0.001	0.49	0.55
SVCs	558,650±126,680[a]	386,800±122,341[a]	2,093,625±277,413[b]	1,845,208±435,608[b]	< 0.001	0.49	0.90
CD45+ cell	321,313±100,559[a]	141,760±43,440[a]	1,057,855±176,466[b]	874,074±231,035[b]	< 0.001	0.29	0.99
Macrophage	93,574±20,182[a]	63,969±20,780[a]	686,589±149,729[b]	534,077 ±141,837[b]	< 0.001	0.44	0.60
NK cell	9,042±1,557[a]	5,656±1,787[a]	53,078±9,462[b]	37,213±10,061[bc]	< 0.001	0.91	0.68
B cell	76,498±46,504	15,064±3,908	42,188±4,978	31,227±8,314	0.68	0.11	0.26
CD4 T cell	44,110±12,968 [ab]	19,166±6,766 [a]	102,932±12,000 [c]	78,041±18,561 [b]	0.001	0.09	0.99
CD8 T cell	25,505±14,113	4,663±1,888	47,306±15,476	57,154 ±23,131	0.04	0.75	0.37

[1]Two-way ANOVA was used to determine the significant effects of fat and vitamin D contents, and an interaction. [ab]Different superscripts indicate significant difference ($P < 0.05$) by Duncan's multiple range test. The data are presented as means ± SEM.
[2]CON: 10% kcal fat diet; HFD: 45% kcal fat diet; DC: 1,000 IU vitamin D/kg diet; 10DS: 10,000 IU vitamin D/kg diet.
[3]Visceral fat includes perirenal, intraperitoneal, epididymal fat.

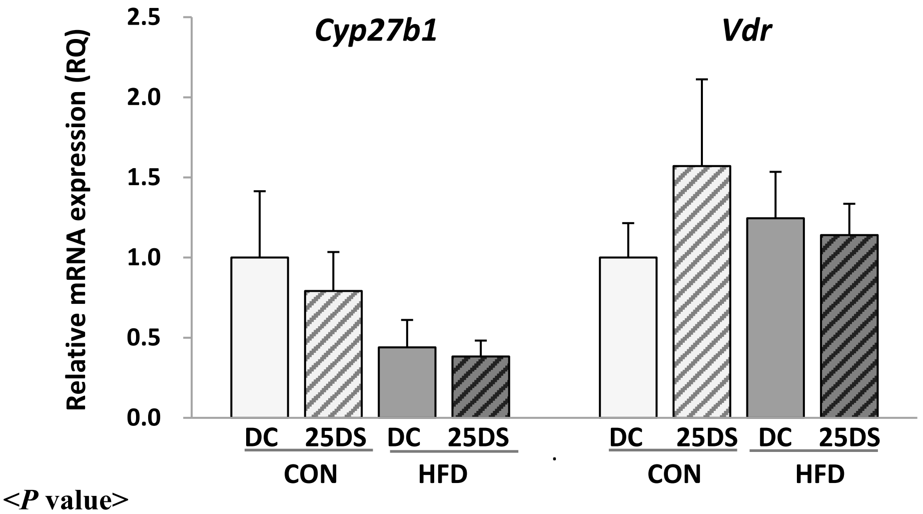

<*P* value>

Fat amount	0.14	0.76
Vitamin D content	0.31	0.45
Interaction	0.43	0.28

Figure S1. The mRNA levels of *Cyp27b1* and *Vdr* in epididymal adipose tissue

Data are presented as mean ± SEM, n = 6~8 for each group. Two-way ANOVA was used to determine the significant effects of fat and vitamin D contents, and an interaction. CON: 10% kcal fat diet; HFD: 45% kcal fat diet; DC: 1,000 IU vitamin D/kg diet; 25DS: 25,000 IU vitamin D/kg diet.

Cyp27b1, cytochrome P450 27B1; *Vdr,* vitamin D receptor.

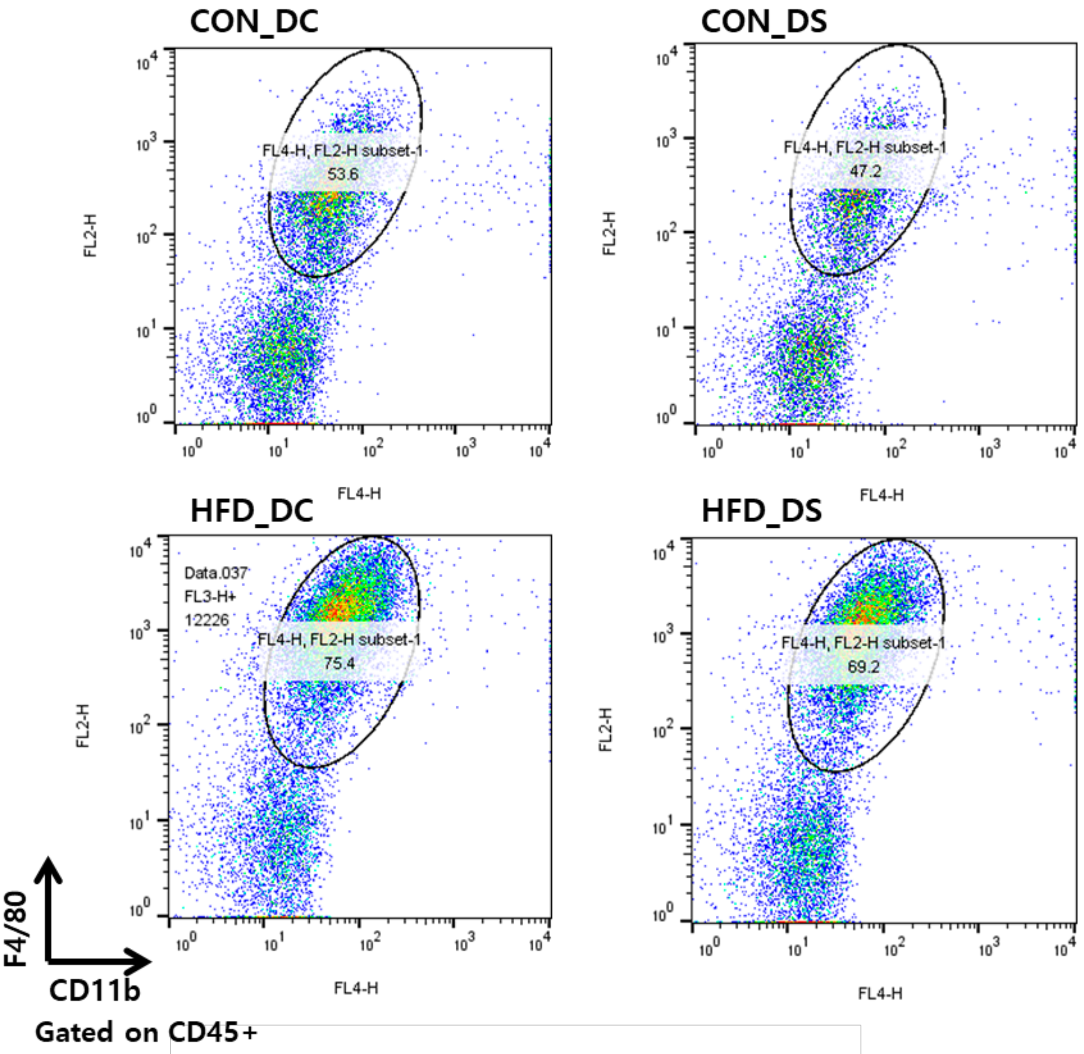

Figure S2. Flow cytometric dot plots of macrophage from visceral adipose tissue
CD45^{+}F4/80^{+} CD11b^{+} cells were presented as dot plots of the FlowJo analyses. CON: 10% kcal fat diet; HFD: 45% kcal fat diet; DC: 1,000 IU vitamin D/kg diet; 10DS: 10,000 IU vitamin D/kg diet.
